# Mouse Models and Online Resources for Functional Analysis of Osteoporosis Genome-Wide Association Studies

**DOI:** 10.3389/fendo.2019.00277

**Published:** 2019-05-07

**Authors:** Robert D. Maynard, Cheryl L. Ackert-Bicknell

**Affiliations:** ^1^Center for Musculoskeletal Research, University of Rochester, Rochester, NY, United States; ^2^Department of Orthopaedics and Rehabilitation, University of Rochester, Rochester, NY, United States

**Keywords:** osteoporosis, mouse models, genetics, bone, functional validation

## Abstract

Osteoporosis is a complex genetic disease in which the number of loci associated with the bone mineral density, a clinical risk factor for fracture, has increased at an exponential rate in the last decade. The identification of the causative variants and candidate genes underlying these loci has not been able to keep pace with the rate of locus discovery. A large number of tools and data resources have been built around the use of the mouse as model of human genetic disease. Herein, we describe resources available for functional validation of human Genome Wide Association Study (GWAS) loci using mouse models. We specifically focus on large-scale phenotyping efforts focused on bone relevant phenotypes and repositories of genotype-phenotype data that exist for transgenic and mutant mice, which can be readily mined as a first step toward more targeted efforts designed to deeply characterize the role of a gene in bone biology.

## Introduction

The NIH Consensus Development Panel on Osteoporosis Prevention, Diagnosis, and Therapy defined this disease as, “*a skeletal disorder characterized by compromised bone strength predisposing a person to an increased risk of fracture*” wherein bone strength was defined as the combination of bone mineral density (BMD) and quality (1). In the year 2000 it was estimated that there were 8.9 million osteoporotic fractures worldwide (2) and existing data suggests that, on average, half of all women and 20% of all men will experience a facture in their adult life (3). The economic burden of osteoporosis is immense, resulting in up to $22 billion in direct health care costs per year in the U.S (4) and €37 billion annually in the European Union (3). Further, osteoporotic fractures are associated with increased morbidity and mortality (5). Bone mineral density, as measured by Dual X-Ray Absorptiometry (DXA), is inversely correlated with fracture risk. For this reason, BMD remains the method used to diagnose this disease clinically. It is estimated that over 50% of the variation in BMD is attributed to genetic factors (6), but importantly in humans, fracture risk is also heritable (7).

Since the first genome wide association study (GWAS) for BMD in 2007 (8), there has been an explosion in the number of loci found to be associated with BMD, bone structure and fracture risk. The largest GWAS conducted to date suggests that there are over 1,000 conditionally independent genetic signals in 515 discrete loci associated with the phenotype of estimated bone mineral density (eBMD) (9). Fracture is inherently a more complicated phenotype, and 14 significant loci were identified in this same study (9). In a second large meta-analysis GWAS, 15 loci were identified as associated with fracture incidence, but all of these loci had been previously found to be linked to traditional DXA derived BMD (10). These data highlight the incredible complexity involved in the genetic regulation of BMD and the difficulties associated with accounting for the genetic regulation of clinically important phenotypes such as fracture incidence.

## The Causal Variant vs. the Candidate Gene

Despite the identification of this astonishing number of loci, these 515 eBMD loci only account for only 18% of the trait variance (9), suggesting that there may yet be more loci to be discovered. Further, one must remain cognizant of the fact that a locus does not equal a mechanism of action. Much of the focus of the so-called “post-GWAS era” is on identifying the underlying gene or genes, pin pointing the causative variant(s) and determining the hows, the whats, the whys, and the whens by which these loci act and interact to cause a phenotype (11). Ideally, every nucleotide in every person would be examined in a GWAS for association between genotype and phenotype. In practice, this is rarely possible due to cost, and fortunately, it is not completely necessary. Over short distances, single nucleotide polymorphisms (SNPs) are often in linkage disequilibrium (LD) with other nearby SNPs (12). It is common practice in GWAS to select representative SNPs or “tagging SNPs” for genotyping, which in turn are used to represent a haplotype (13–15). This tagging genotyped SNP is a proxy for the causative variant and may or not have any functional role in disease.

Overwhelmingly, the causative variant for a given genetic locus is not located within the coding region of a gene, and even more rarely is the causative variant one that leads to an altered protein product. Rather, causative variants are often located in intergenic regions and are thought to modify the expression of one or multiple genes (16). Thus, the term “causative variant” is not to be confused with, nor is it synonymous with, the term “causative gene” (11). Understanding the nature and mechanism of action of the causative variant is critical for understanding the etiology of disease. A case in point is the comparison of two Mendelian conditions: Van Buchem disease and Sclerosteosis Type I. In both of these conditions, a thickening of the cortical bone, narrowing of the medullary canal of the long bones and thickening of the mandible are observed (17, 18). However, gigantism is seen in Sclerosteosis (17), but not in Van Buchem disease. In Van Buchem disease, a 52 Kb deletion occurs in an intergenic region on human Chromosome (Chr) 17q21.3 (19) and putatively impacts expression of two genes: *MEOX1* and *SOST* (20). For Sclerosteosis, up to 10 homozygous loss-of-function mutations in the coding region of the *SOST* gene have been identified (21). Thus, in both of these diseases, there is a common gene impacted, but clinically the presentation is different, in part because the causative variant(s) leads to disease in differing ways.

Following this same theme, functional validation of GWAS candidate genes is not to be confused with the identification of the functional variant(s). The functional validation of a candidate gene means to determine if that gene could plausibly be associated with the phenotype of interest. Both functional validation of a candidate gene and determination of the causative variant are of value for understanding human disease especially when there are one or more uncharacterized genes in the locus (22). To be a candidate, a gene must fulfill two straightforward criteria. First, the gene must be expressed in the appropriate tissue(s) and at an appropriate time point to influence the phenotype of interest. Second, the gene must play a role in a biological process relevant to the phenotype of interest (11). For many diseases, the first criteria can be used to remove a surprising number of candidate genes and is therefore an easy first pass filter to narrow down to genes of interest. However, for bone, what constitutes an appropriate tissue or appropriate time point is less easy to define, yet is critical for the design of experiments to determine function (11). The reasons for this are that bone turnover, bone size and geometry, BMD and even fracture risk, are impacted indirectly by a number of other organ systems such as the digestive tract (23), brain (24), kidney (25), and skeletal muscle (26), and processes occurring during development that have lasting impacts on the adult skeleton (27). That said, the majority of validated GWAS genes impacting BMD appear to be expressed in bone tissue (9, 28). The second criteria, namely that the candidate gene plays a role in a relevant biological system, can be a little harder to ascertain, especially for uncharacterized or understudied genes for which there is little known about function. It is here that the mouse has proven to be invaluable (22), and indeed, the bulk of functional validation has been accomplished by so called reverse genetic approaches in mice.

## The Genome of Mice and Man

Mice have been used for over 100 years to study the genetic regulation of physiology, development and disease (29). Like other animal models, mice fill two specific needs particularly well: they can be used to collect phenotype data that cannot be collected from human subjects, and they can be used to study single factors (i.e., diets, alleles, ages) in isolation. The mouse genome, while smaller than the human genome, is highly conserved for protein coding genes (22). At the gene level, ~17,094 mouse protein coding genes have a known direct human ortholog (http://www.informatics.jax.org, accessed Oct 2018), and overall organization of the mouse and human genomes is remarkably syntenic despite 75 million years of evolutional distance between the two species (30). Thus, genetic findings in mice are often concordant with genetic findings in humans (31). However, with the refinement of GWAS and improved annotation of the human genome, data is accumulating to suggest that long non-coding RNA genes also play a role in human disease (32) and not surprisingly, these non-coding genes have been found at GWAS loci for bone phenotypes (33). While homologs for long non-coding RNA genes have been found in mice for human genes (34), generally, these genes are poorly conserved (35).

## Differences Between Mouse and Man in Bone

The physiologic and anatomic similarity between mice and humans has long been appreciated, and, given the high degree of genome homology, is not surprising (31). Regardless, there are differences in the skeletal system that should be considered in a functional validation experiment. In mammals other than mice, lamellar bone is organized into Haversian systems or secondary osteons in which lamellar bone is arranged in concentric rings around a central cavity (36) whereas in mice, a Haversian system of organization is not seen (37). There are also subtle differences between mouse and human bone growth during aging. In humans, the epiphyses fuse shortly after puberty resulting in a halt in long bone growth. In mice, epiphyseal fusion either never completes or is delayed until old age (depending on the strain), and thus some strains of mice can continue to experience some degree of long bone growth to at least 2 years of age (37). In both men and women, cortical bone gain has essentially stopped in early adulthood, and a steep loss in cortical bone volume begins at menopause in women and after the age of 75 years old in men. In contrast, trabecular bone loss begins in early adulthood, irrespective of sex (38). In mice, cortical bone volume increases out to at least 7 and possibly 12 months of age (depending on strain), in part due to the increases in skeletal size that arise from continued growth (39, 40). Decreases in trabecular bone amount occur far sooner in mice than in humans. In fact by weaning, inbred mice have already begun to lose trabecular bone (41) and in outbred mice, a complete lack of trabecular bone volume in the distal femur was observed as young as 6 months of age (42). In comparison to humans, this would be the equivalence of bone loss beginning in toddlers to the point of complete loss of trabecular bone in some anatomic sites as young adults. The aggregate peak femoral volumetric BMD in mice (cortical and trabecular) is generally accepted to happen at about 16 weeks of age, but this varies by mouse strain (39). This does not mean that mice are inappropriate for the functional validation of human bone GWAS loci, but rather that experiments must be designed to ensure that appropriate comparisons in bone are being made. Justifying an age for mice in a functional validation experiment is not as simple as scaling chronological age relative to lifespan and calling it equivalent.

## Reverse Genetics

Reverse genetics simply means to reverse engineer the function of a gene in a biological system. In contrast, forward genetics approaches such as GWAS move from a disease or phenotype to find the genetic cause. Thus, reverse and forward genetics are inseparable for the study of human disease (22). The mouse genome is easily modified, and 62,025 targeted alleles in 16,947 genes are listed in the Mouse Genome Database (http://www.informatics.jax.org/, accessed October 2018). This means that for some genes, multiple targeted alleles have been constructed and at least partially characterized but for a fraction of protein coding genes, we as of yet have no direct evidence of function gleaned from genetically engineered mouse models. The methods for generating these targeted mouse models are described in detail elsewhere (43), but what these models are and where to find both the mice and the phenotype data available for these mice is described in greater detail in the sections below.

Both global and tissue specific models have been used to functionally validate bone GWAS loci. An elegant example using both global and cell type specific models in mice is the work conducted to confirm the *WNT16* gene as a candidate gene for bone mass and fracture risk (44). In this study, the authors used both global and cell lineage specific knockout mouse models to show that WNT16, a secreted factor, is produced by the osteoblast and acts on the osteoclast precursor to inhibit osteoclastogenesis. In addition, this WNT also acts on the osteoblast to inhibit the formation of the osteoclastogenesis inhibitor Osteoprotogerin (OPG). As a result, the loss of the *Wnt16* gene globally in mice or in the osteoblast lineage only results in an increase in osteoclast-mediated bone resorption leading to reductions in cortical bone mass, but interestingly not loss of trabecular bone. Further, these mice present with spontaneous fractures of the long bones, a phenotype rarely seen in laboratory mice. Thus, this study confirmed that *WNT16* is indeed a bona fide bone gene and was able to demonstrate the mechanism of action by which fracture risk was increased.

A global knock out may not be desirable or plausible for the study a gene in adult bone biology. A case in point is the global *Runx2* knockout mouse, which dies shortly after birth presumably due to breathing difficulties (45). As is outlined in the *Wnt16* example, conditional knockouts and inducible knockouts allow one to restrict gene loss to a cell type of interest and/or after, critical development milestones have been met. Such studies require an appropriate Cre-diver strain wherein expression of Cre-recombinase is restricted to a desired cell type and/or time point in cell maturation. Ideally, this allows excision of the gene of interest in only the cell type of interest. Some of these Cre-drivers are inducible, meaning that the timing of Cre-induction can be carefully controlled. A summary of many of the bone relevant Cre-Driver strains for musculoskeletal tissues and cells that have been described in the literature are summarized here [reviewed in Elefteriou and Yang (43)]. In addition, several Cre-databases are available online which provide more up to date information about where the Cre-driver is expressed (**Table 2**). It is important to remember that Cre-drivers may be expressed in undesired tissues as well as the desired location.

## Phenotyped Mouse Models

The nascent stages of the identification of mouse models of human disease relied on the identification of outliers in a colony of mice, followed by breeding to determine heritability of the observed phenotype (46). With advances in technology, the process of finding the mutation(s) causing the phenotype has changed, but finding spontaneous mutations in mice remains a valuable source of human disease models. Many spontaneous mutations are not gene ablation models and may more closely mimic human disease than a knockout mouse (46). Relevant to bone biology are models such as the *oim* mouse, which was discovered in a breeding colony at the Jackson Laboratory in 1985. In this mouse, a single base pair deletion in the *Col2a1* gene results in a truncated protein product (47), and phenotypically this mouse mimics aspects of Osteogenesis Imperfecta Type III (48).

A second method to generate mouse models of human disease is chemically induced mutagenesis via delivery of compounds such as Ethylnitrosourea (ENU) (46). This forward genetics approach, while successful in that many models for various diseases were generated, is laborious and inefficient as the location of the mutation(s) is random in the genome and therefore genes impacting the phenotype of interest will not be specifically targeted. While it is possible to identify recessive traits in an ENU protocol, it is much faster to restrict a screen to find traits acting in a dominant fashion. Typically, so-called Generation-0 (G0) male mice are treated with ENU to induce mutations. The G0 males are bred to wildtype females to generate so called G1 offspring, which are then screened for phenotypes of interest. Approximately 2–4% of these G1 mice will carry mutations yielding a phenotype (49). Several models relevant to bone biology have been identified this way (50–54). For example, we recently described the *tvrm111B* mutant mouse strain wherein an inactivating mutation in *Lrp5* was identified. As expected, these mice have mild decreases in bone mass, abnormalities in the retinal vasculature and other eye phenotypes, and are a model of osteoporosis pseudoglioma (OPPG) (55).

With the completion of the first draft sequence of the mouse genome in 2002 (30), sights were set on determining the function of all of the known and newly discovered genes. By this time, generating genetically engineered mice was common practice and becoming increasingly more efficient (49). This resulted in the development of two “mouse clinics” pilot programs to make new models of human disease: the Mouse Genetics Project (MGP) at Sanger in the UK and the multi-site European Mouse Disease Clinic (EUMODIC) program (56). In short, *de novo* transgenic knockout mouse models were generated, and this was coupled with the employment of high throughput, comprehensive and cost effective phenotyping pipelines to characterize these new strains. These projects largely were designed to be hypothesis free in that the genes of interest were not pre-screened to be involved in a specific disease. The goal of these clinics was 2-fold: (1) to identify new models of human disease and (2) catalog the function of protein coding genes in the mouse. These mouse clinics enjoyed economy of scale allowing for more phenotypes to be captured per animal than was previously possible in a single laboratory working in isolation (57).

The mouse clinic method identified weaknesses in the gene-by-gene study approach that had been the mainstay of determining mammalian gene function. From these preliminary proof-of-concept mouse clinics it became apparent that pleiotropy is very common, yet commonly new mouse models were only being phenotyped for traits relevant to the interests of the group making the model. This observation of pleiotropy led to the concern that incomplete information was being generated in the historical gene-by-gene approach. Further, there was concern that that inconsistent data was being collected, as the gene-by-gene approach was not held to any standardized methods for data collection (56). In contrast, the application of a systematic and high-throughput phenotyping pipeline overcame these issues wherein only “some” types of data were collected per strain and allowed enforcement of data collection standard operating procedures (SOPs) (56). Another major issue with the gene-by-gene approach is that mouse models were generated and/or maintained on a wide variety of genetic background strains, precluding straightforward comparison of one model to another because of strain background differences. Further, breeding of one model to another created the risk of passenger mutation effects (58). With all of this in mind, the “second generation mouse clinics” were carefully designed with standardized and validated SOPs developed for both animal model generation and for capturing the phenotype data. The phenotyping pipelines and the SOPs for collecting data are reviewed extensively elsewhere (59), but below some of the pros and cons of the largest of these data collections are described in the context of validating GWAS loci for bone phenotypes and disease. Table 1 summarizes these data collections.

**Table 1:** Selected repositories of phenotyping data for mouse genetic models.

**Title**	**URL**	**Content**
BoneBase	http://bonebase.org	In-depth bone specific phenotype data for selected IMPC generated mice.
International Mouse Phenotyping Consortium	http://www.mousephenotype.org/	The website of the IMPC, including SOPs, data, and resources for ordering IMPC mice and targeted ES cells.
Origins of Bone and Cartilage Disease	http://www.boneandcartilage.com/	In-depth bone specific phenotype data for selected IMPC generated mice.
Mouse Genome Database	http://www.informatics.jax.org/	The international resource database for the mouse. Includes genomic, phenomic and gene function information.
Infrafrontier	https://www.infrafrontier.eu	Access to mouse models and data collected by mouse clinics in Europe and Canada. House the European Mouse Mutant Archive (EMMA).

### International Mouse Phenotyping Consortium (IMPC)

The International Knockout Mouse Consortium (IMKC) began in 2003 with the goal of making embryonic stem cells carrying a knockout allele for all protein coding genes. Indeed, embryonic stem cell (ESC) lines carrying mutant alleles were generated for 18,500 genes (60). This effort was conducted by numerous sites and programs internationally, including the Knockout Mouse Program (KOMP) in the United States. The vast majority of these mutant alleles are knockout–first and conditional-ready, meaning that by employing appropriate breeding strategies, both global gene ablation can be achieved or genes can be knocked out in a temporal or cell/tissue specific manner (61). It must be noted, though, that not all genes were knocked out in this fashion. A smaller fraction of the ESC cell lines are knockout-only (22). There many impressive aspects of this ambitious and highly successful project, but the one that is perhaps not as well appreciated by non-mouse geneticists is that all of these cell lines were created on a single genetic background, C57BL/6N (60). As a result, when animated into live mice, double- and triple-knockouts can be generated without the time consuming and costly step of breeding all lines onto a uniform genetic background before interbreeding (58). In 2011, the International Mouse Phenotyping Consortium (IMPC) was formed to conduct high-throughput, multi-systems phenotyping on the IMKC generated mice. In 2015, the efforts of the IKMC were folded into that of the IMPC, and, under the umbrella of the IMPC, mouse model generation continues. It should be noted that the use of CRISPr/Cas9 is becoming more widely adopted by the IMPC, producing global gene disruption including conditional and lacZ reporter lines. However, like the previous mutant alleles, these new models are being made on the C57BL/6N background (59).

Currently, the IMPC is comprised of 19 research institutions located in 11 countries and was funded by five national funding organizations. For the 10 year span that the IMPC was funded to operate (2011 to 2021), five goals were laid out: (1) create a consortium capable of generating targeted mutations for 20,000 mouse genes, (2) conduct high-throughput, standardized phenotyping of these knockout lines, (3) determine the biological function of these genes, (4) create a network of secondary phenotyping consortia that can conduct additional phenotyping to enrich the primary data set, and (5) provide the means and support for free and unrestricted data disseminations for all IMPC generated data (56).

At the heart of the IMPC is the phenotyping pipeline (https://www.mousephenotype.org/impress/). This pipeline can be divided into four sections. In the first part, lines are assessed for viability and fertility in the homozygous global knockout state. Approximately one third of all IMPC knockout lines generated to date were found to be embryonically lethal (no homozygous knockout mice found after screening 28 pups from a heterozygous by heterozygous mating) or sub-viable (less than half of the homozygous knockout mice survive to weaning) (62). In recognition of this high number of non-viable lines, an embryonic pipeline is currently in development. This pipeline is envisioned to collect the duration of viability post fertilization, and histopathology and gross morphology data at multiple time points during development. In the third part of the pipeline, a robust set of phenotype data is collected covering most body systems. This adult phenotyping pipeline has been applied largely, but not exclusively, to homozygous knockout mice. This pipeline is conducted using a rigid schedule of tests starting when the mouse is 9 weeks of age and extends until the animal is euthanized at 16 weeks of age. This test battery consists of a core set of 15 tests that are conducted at all phenotyping sites using carefully developed SOPs, as well as a set of optional tests that are collected at some, but not all, of the phenotyping centers. Lastly, at euthanasia, biological specimens are collected and analyzed. Like for the *in vivo* testing, there is a core of data collected on all mice as well as optional collection SOPs (56, 59). For example, all sites must collect data regarding heart weight at death, but only some of the sites bank tissues and embed them for histopathology (59).

There are two sets of data collected on mice in the IMPC pipeline that are of primary interest to bone biologists: body composition and skeletal dismorphology (56). Body composition traits, including bone mineral content (BMC), bone area (BA), bone mineral density (BMD), lean mass and fat mass, are collected. All of these phenotypes are collected on the whole body sans the head via Dual X-ray Absorptiometry (DXA) on male and female mice at 11 weeks of age. At the same time, a simple 2D whole-body X-ray is collected, and a very comprehensive list of bone sites are examined for malformations and dismorphologies (57). The way this pipeline is set up, data is collected on each line until 7 males and 7 females per line have been examined for body composition and at least 5 males and 5 females have had X-ray images captured (https://www.mousephenotype.org). Control mice of the C57BL/6N line are run through the pipeline such that a new cohort of control mice is started through the pipeline every week and therefore, there is always concurrent control data collected for every mutant strain. The data for each mutant strain is compared to the aggregate collection of control data using a statistical analysis protocol designed to be robust to the imbalance of group sizes between the cases (mutants) and controls (63). The data is presented on the IMPC web-portal and can be screened in a number of ways. For example, an investigator can look specifically for the BMC data for their favorite strain only, search for all lines with significantly higher BMD and they can download the raw data for their own analyses.

There are many advantages of using these data for functional validation of GWAS loci. As of data release 8.0, which was announced on July 16th of 2018, phenotype data for 5,115 genes were available, which is just over 20% of all known protein coding genes in the mouse genome (https://www.mousephenotype.org). This data is freely available for use by anyone at any time and is presented in an easy-to-interpret format on the IMPC website. Further, this data can be downloaded and queried in bulk allowing one to quickly search their list of GWAS candidate genes for those with a known bone mass phenotype. At the time of writing this review, just under 300 lines (6.4% of all those tested) were annotated to have an abnormal BMD or BMC phenotype (http://www.mousephenotype.org, accessed October, 2018). Equally important, this list can be screened to eliminate genes that were tested and found not to impact any of the bone phenotypes examined. This latter step can be critical when more than one candidate exists for a single locus. Lastly, the mouse can be ordered from the IMPC to conduct additional phenotyping should an investigator choose.

There are many caveats and cautions that must be considered when using this data for functional validation. In the 7 years since the start of the IMPC, technology has advanced. There is now data available in the IMPC database from multiple different DXA scanners that range in resolution from ~180 μm spatial resolution for the older (and now no longer commercially available) PIXImus scanners made by GE-Lunar® to ~50 μm spatial resolution for the newer instruments from Faxitron®. While both instruments have been validated against bone ash weight standards, the superior resolution of the newer instruments may provide increased fidelity in BMD via refinement in accuracy of projected area measured (64). As a result, there will be less noise in measures such as bone area, which may or may not affect achievement of statistical significance for any mutant line.

DXA BMD in a mouse is different than that collected usually for GWAS purposes in humans. Even with the superior resolution of the newer DXA machines, in mice, these instruments are not able to discriminate between cortical and trabecular regions of interest without specialized analysis (65). It has been estimated for long bones that three quarters of the bone mass is contributed by the cortical compartment primarily in the diaphyses (66). The majority of the attenuation of the X-ray in DXA imaging for the whole body of a mouse is achieved by the cortical compartments (65). However, BMD for clinical purposes is measured in the lumbar spine, which is largely trabecular, as well as in the hip, which is proportionally more trabecular than the femoral diaphysis. While anatomic site-specific region of interest (ROI) data can be captured on the mouse DXA instruments, this data is not typically available in the IMPC database. Phenotypes that impact bone in subtle ways, such as only in the trabecular compartment or in only one anatomic site, may be missed by the IMPC screen. In this scenario, the mouse line could be mistakenly annotated as having no abnormality in bone mass.

All of the bone phenotyping in the IMPC pipeline is collected on 11 week old animals. From a sexual maturity point of view, this represents an adult animal, but from a skeletal growth point of view, these mice are still in the bone acquisition phase. As was outlined earlier, cortical bone volume can increase far past this 11 week age point (40). Thus, these 11 week old mice would likely be the equivalent of an adolescent human. It could reasonably be argued that the trends leading to lower/higher adult BMD or smaller/larger adult skeletal size will be well established by 11 weeks of age, but one should remain cognizant of what these data represent when using it to interpret and functionally validate a human GWAS locus.

While Quantitative Ultrasound (QUS) phenotypes do moderately correlate with areal BMD (67), bone architecture, and mechanical properties in humans and large animals (68), there is no directly measurable equivalent phenotype in mice for speed of sound (SOS) or Broad Ultrasound Attenuation (BUA). Because of the relationship between estimated BMD (eBMD) as determined from ultrasound measures and areal BMD from DXA (67), it is assumed that the same advantages and caveats for using IMPC data for functional validation of areal BMD GWAS loci also apply to eBMD loci. Similarly, IMPC does not contain equivalent data such as trabecular bone volume and other compartment-specific phenotypes like those captured by the ultra-high-resolution CT machines. Therefore, while the IMPC is a rich source of data, it may not have utility for functional validation for some GWAS.

As mentioned previously, the IMPC mice are generated on a C57BL/6N (N) genetic background (62). This strain is related to, but is not genetically the same as the more commonly used C57BL/6J (J) strain or other C57BL/6 strains available from other vendors. Over 200 generations of breeding have occurred since the J and the N lines were originally separated in 1951 and during that time, almost 700,000 genetic differences have accumulated between these two strains including 51 coding variants (69). It is not surprising that phenotypic differences between these strains have been observed including differences in behavior, blood pressure, metabolism and immune function. In direct comparisons of the two, it was observed that the N males and females have higher body fat whereas the J strain has increased lean body mass. The male J mice trend toward increases in whole-body BMD, but no differences in trabecular bone mass or bone turnover markers were observed for either sex (69). It is well understood that changing genetic background can modify the phenotype of knockout and transgenic mice due to modifier genes, complicating the interpretation of the impact of a gene on a phenotype (70). Direct comparisons on a uniform genetic background takes multiple generations of backcrossing to avoid effects from segregating modifiers (71). This matters for functional validation of GWAS loci as segregating modifiers present on a mixed genetic background can mask or alter the phenotypic presentation of the allele of interest, leading to inappropriate conclusions about a gene's involvement in a biological system of interest.

### BoneBase

Two programs are expanding the skeletal phenotypic data available for IMPC mice. The first of these, BoneBase, is located in the US. This program received live breeder mice from The Jackson Laboratory IMPC production site to conduct in-depth skeletal phenotyping. It should be noted that this program was not part of the IMPC and received independent funding but did work with the IMPC data coordinators. Like the IMPC, this program was designed to be hypothesis-free in that lines were not a priori selected because of evidence suggesting a role in bone biology. All lines that were viable, fertile and free of profound pathologies (i.e., early renal failure, spontaneous early cancers, etc.) were accepted for this program. This program was designed to add on to, but not replicate, the data generated by the IMPC (72).

**Figure 1:**
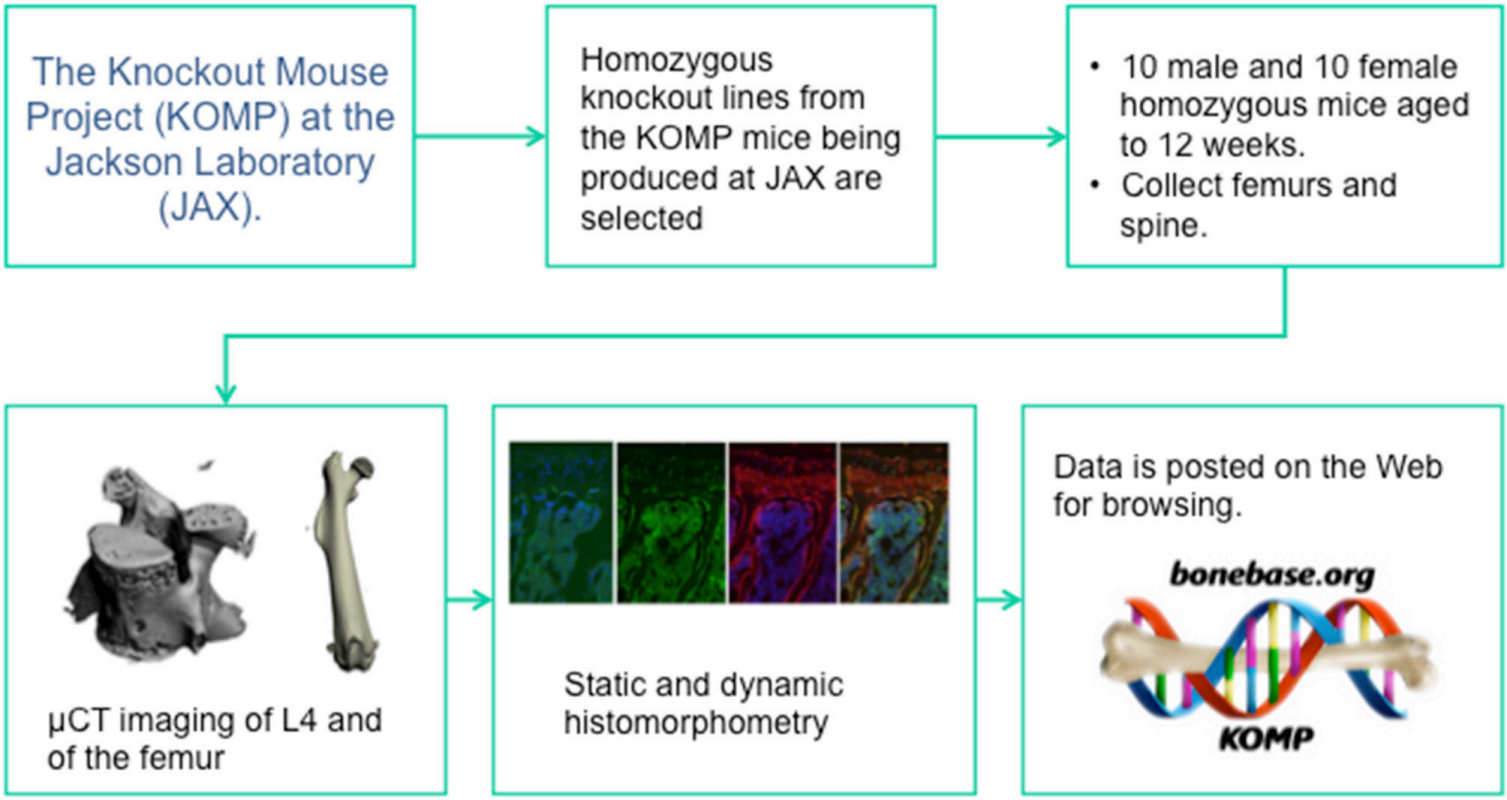
The data collection pipeline for the BoneBase.org phenotyping project. This is one of two specialized high throughput phenotyping pipelines that is conducting auxiliary, bone specific phenotyping of mice generated by the IMPC. The Bonebase.org logo is used with permission from the database owners.

Like the IMPC, homozygous animals were used for phenotyping. Group sizes of at least 8 male and 8 female mice were phenotyped at 12 weeks of age. Two main phenotyping mechanisms were used: microCT analysis of the lumbar vertebrae and the femur, and dynamic cryo-histomorphometry (73) of adjacent lumber vertebrae and the contralateral femur. The pipeline (Figure 1) was set up such that if a phenotype at either anatomic site or in either sex was found, cryo-histomorphometry was conducted and this data is not available for all lines. In this manner, a wide-ranging set of data was collected capturing information on cortical bone size and shape, trabecular bone mass and architecture, bone formation, and osteoblast and osteoclast number. Also like the IMPC, rigorous SOPs were implemented at all stages of animal breeding, tissue collection, and analysis to ensure that data was collected in an unbiased and rigorous fashion. Also like the IMPC protocol, this group collected data from C57BL/6N mice at regular intervals to ensure that concurrent controls existed for every line, however, these controls were collected monthly, not weekly (72).

**Figure 2:**
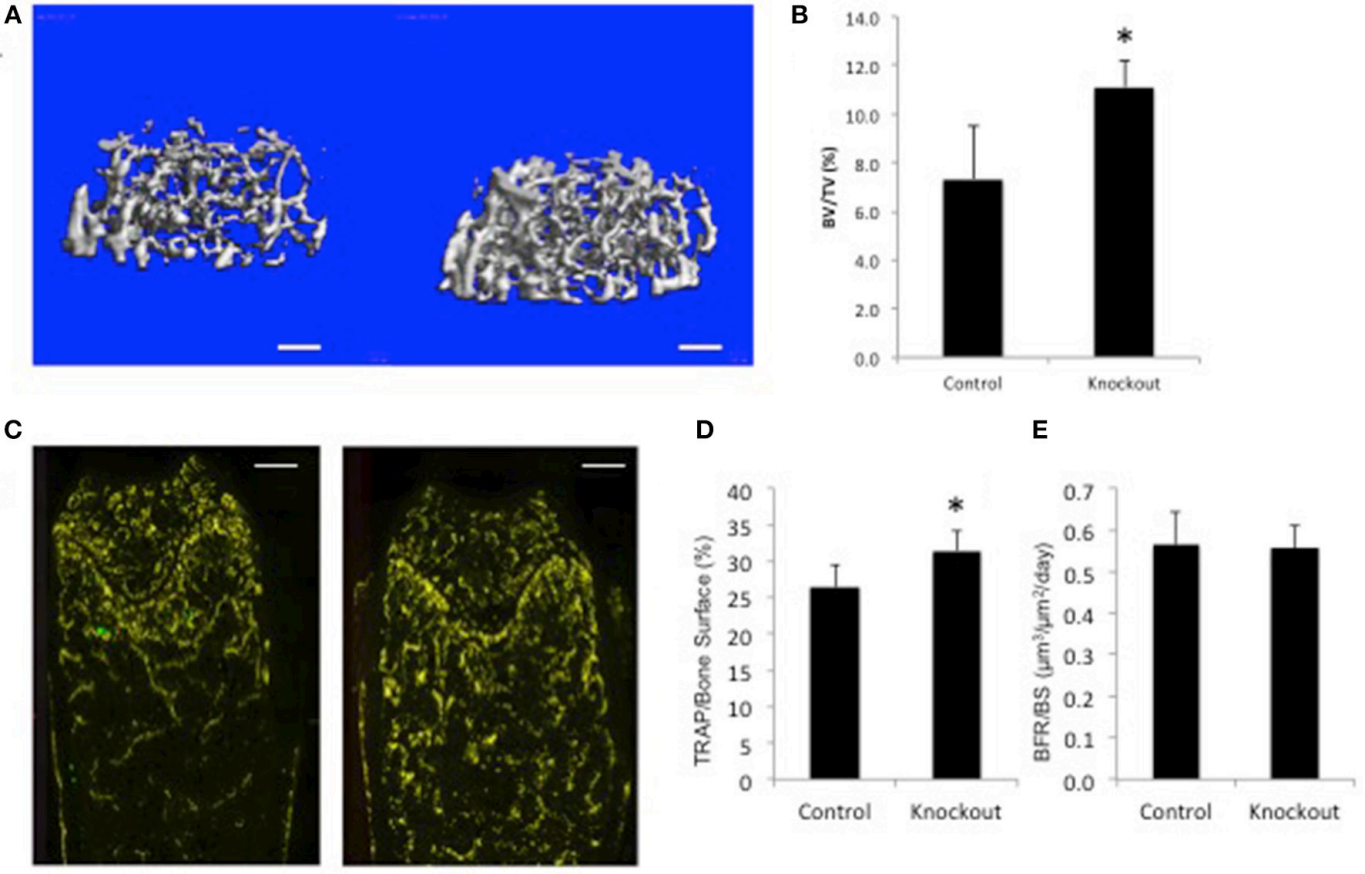
Bone Phenotype of female mice lacking *Ocstamp*. Representative data as collected via the BoneBase pipeline for Ocstamp null female mice. **(A)** Reconstructions of the distal femoral trabecular compartment for *Ocstamp*^+/+^ (left) and *Ocstamp*^−/−^ mice. **(B)** A significant increase in bone volume over total volume (BV/TV) was observed in the null vs. control animals (^*^p < 0.001). **(C)** Staining for tartrate resistant acid phosphatase (TRAP), a marker of osteoclast cells (yellow) in *Ocstamp*^+/+^ (left) and *Ocstamp*^−/−^ mice. **(D)** An increase in the number of TRAP positive cells per unit bone surface observed in the null vs. control animals (^*^P = 0.001), but no difference in bone formation rate (BFR) was seen **(E)**.

For illustration purposes only, the data for a single gene examined in the BoneBase pipeline (Figure 2). The data presented here, which is freely available at the BoneBase web portal (Bonebase.org, accessed Oct, 2018), is for the gene *Osteoclast stimulatory transmembrane protein* (*Ocstamp*), which is not a known GWAS candidate gene. This gene is part of a growing list of genes shown to be required for the fusion of pre-osteoclasts into mature multinucleated and functional osteoclasts (74). A substantial increase in bone volume fraction (BV/TV) in the femur (Figures 2A,B) was observed in the female but not male mice (data not shown). A substantial increase in the amount of TRAP staining per unit bone surface (TRAP/BS, Figures 2C,D) but no change in bone formation rate (BFR, Figure 2E) was noted. Collectively, these data suggest an involvement of the osteoclast, but not the osteoblast. From this simple example, it is readily apparently how this is a valuable resource for functional validation of GWAS loci as information is available to provide confirmation that a gene impacts bone biology. In addition, putative mechanistic information is also available to provide a first tier of evidence about how a candidate gene at a locus acts to impact bone biology without the costly investment in *de novo* model construction.

It is interesting to note that while the IMPC found that 6.4% of lines presented with a bone phenotype by DXA alone (www.mousephenotype.org), the Bonebase protocol found that ~15% of all lines presented with an increase or decrease in bone mass as determined by microCT. There was little overlap between those determined to be bone genes by the IMPC and those found by the BoneBase protocol (72). Given that whole body areal BMD obtained from DXA is largely a cortical and bone size driven phenotype (65) and that microCT can be used specifically to look only at the trabecular bone, this is not an unexpected finding. This is further supported by evidence that suggests that trabecular and cortical bone are controlled by independent genetic signals (75–77).

All of the data generated by the Bonebase project can be queried at any time via a webportal (www.bonebase.org). To date, 220 lines have been analyzed and the data for these lines is available both as summary statistics for a line (separated by sex) and as raw data available for each individual mouse (as is presented in Figure 2). It is interesting to note that far more anatomic site-specific effects (i.e., only in the spine or only in the femur) and sexually dimorphic effects (i.e., only in males or only in females) were found than that which has been noted for genetic loci in GWAS. This may reflect differences in mice vs. humans, or may reflect that subtle effects could be more easily detected in this repeated measures study design.

### Origins of Bone and Cartilage Disease Project

The Origins of Bone and Cartilage Disease (OBCD) project is the second of two programs expanding skeletal phenotype data (57, 78) and is very similar in philosophy to that of the BoneBase project. Like BoneBase, this project is designed to expand on the phenotype data collected on IMPC generated mice. This project uses mice generated by the Welcome Trust Sanger Institute (WTSI) IMPC phenotyping pipeline and, to date, data is available for 733 lines. The summary data is available for all lines examined by the OBCD and is provided in a straightforward web portal (http://www.boneandcartilage.com/index.html). Unlike the Bonebase project, OBCD was able to collect bone samples from the same mice that went through the primary IMPC phenotyping pipeline and these mice are 16 weeks of age at phenotyping. A primary difference between these two programs is that only data from female mice are available for the traits of interest for bone and osteoporosis research in the OBCD, but data exists for males and females in Bonebase. Since the OBCD collected samples directly from the WTSI pipeline, data is available for some lines in a heterozygous state (57). This may be a better reflection of what is captured by GWAS, as many GWAS loci do not negate expression or alter protein function. The haploinsufficient state may more closely mimic what is expected to be the consequence of many GWAS loci.

There is some overlap in the types of data collected by these two projects, but each project has a different focus with regards to the kind and purpose of the data collected. Both groups conducted microCT-based imaging of the distal femur and femoral midshaft and both groups report data on trabecular bone mass and architecture, as well as cortical size and geometry (57, 72). However, the OBCD group collects two types of data that are unique to this program. First, they collect digital X-ray microradiography on the femur and caudal vertebrae to collect bone mineral content (BMC) data. This method overcomes some of the limitations already outlined regarding the IMPC DXA data (57). This method is site specific, has higher resolution than the older DXA machines, and there are no concerns about artifacts arising from extra-osseous calcification. Second, measures of bone strength and stiffness are collected by the OBCD via mechanical testing of the femur (via three point bending) and the caudal vertebrae (via compression). In addition to the bone data described above, this group has plans to collect a plethora of data related to the knee joint which may be informative for osteoarthritis (79). This arm of the project uses the male mice generated by the WTSI pipeline, but data for only 29 strains is currently available.

### Lexicon Pharmaceuticals Inc.

Between 2000 and 2008, Lexicon Pharmaceuticals Inc. embarked on an ambitious project to generate and phenotype ~5,000 knockout mice via a high throughput pipeline. The overarching goal of this project was to identify novel avenues of therapeutic intervention for a wide variety of diseases. The choice of genes for interrogation was enriched for enzymes, receptors, and secreted proteins (80). To find genes of interest, their phenotyping protocol was designed to capture information on behavior, cardiology, immunology, metabolism, oncology, and ophthalmology. Of note for bone biology, three types of data were collected: (1) DXA imaging, including whole body and region of interest (ROI) analysis of the femur and spine, (2) microCT imaging of the fifth lumbar vertebrae and femoral midshaft and (3) static histological analysis of the long bones (80). In total, bone relevant data was collected for 3,762 genes; however, the complete DXA and microCT analysis was not conducted on all lines. This program did identify and name 10 novel genes that are involved in the regulation of bone. An additional three genes were identified as having a role in bone biology, but as of yet the names of these genes are being withheld. Lastly, they confirmed the role in bone biology for an additional 23 genes (80). A subset of the data generated by this project can be found on the MGI webpage (http://www.informatics.jax.org/knockout_mice/).

### Mouse Genome Database

The mouse genome database (MGD) is maintained at the Jackson Laboratory (http://www.informatics.jax.org (81), and is a central part of the larger Mouse Genome Informatics (MGI) consortium. The MGD is an incredible resource for the study of the mouse as a model of human disease and serves as the repository for information regarding mouse genes, gene function(s), and mouse strain information. At present, it contains a summary of the phenotype(s) associated with over 50,000 mutant alleles in over 12,000 genes (http://www.informatics.jax.org, accessed, Oct, 2018) Unlike the resources listed above, the MGD is not, in and of itself, making and phenotyping new mice. Rather, the data contained in the MGD comes primarily from the literature and is entered by expert curators. However, data from other sources such as the IMPC is captured. All of the data presented in the MGD is linked to the primary references and to other mouse model resources. A summary page for each mouse gene is provided and included on this page are: the human homolog, any human diseases associated with that gene, a brief synopsis of the phenotype of knockout mice or mice carrying mutations in that gene, and a visual presentation of the physiological systems affected by mutations in this genes (81). In addition, the MGD and the parent MGI project (82) have built an ever-increasing toolbox for mining this data collection. While it is possible to bulk query this data set for terms such as “decreased trabecular bone mass,” more complete information is obtained by searching for each gene individually when looking to validate GWAS candidate genes. In Table 2, the links for selected search engines and databases useful for finding mouse strains are provided.

**Table 2:** Selected resources for locating inbred, transgenic and mutant mouse strains and targeted ES cells.

**Name**	**URL**
International Mouse Strain Resource	http://www.findmice.org/index.jsp
Australian Phenomics Facility	http://pb.apf.edu.au/phenbank/homePage.html
Canadian Mouse Mutant Repository	http://www.cmmr.ca/
European Mouse Mutant Archive	https://www.infrafrontier.eu/
International Mouse Phenotyping Consortium	http://www.mousephenotype.org/
Riken Bioresource	http://mus.brc.riken.jp/en/
Charles River	https://www.criver.com/
The Jackson Laboratory	www.jax.org
Taconic Bioscience	https://www.taconic.com/
Envigo	https://www.envigo.com/
NIH Aged Rodent Colonies	https://www.nia.nih.gov/research/dab/aged-rodent-colonies-handbook
International Gene Trap Consortium	https://igtc.org/
MGI Cre portal	http://www.informatics.jax.org/home/recombinase
NCBI guide to mouse genome resources	https://www.ncbi.nlm.nih.gov/genome/guide/mouse/

## Transcriptomics

The integration of –omics data such as transcriptomics has been highly successful in many areas of research for identification of the causative variant(s) as well as for interpreting the role of a causative variant and/or candidate gene in the disease process. It is difficult to collect large numbers of specimens from humans for bone research, which limits the number of sizeable expression resources available for human tissue (57). Further, extracting quality RNA from bone and cartilage is laborious and technically challenging (83). A large number of databases containing raw and processed gene expression data exist. The largest of these are described below and summarized in Table 3.

**Table 3:** Selected resources for gene expression and localization in the mouse.

**Title**	**URL**	**Description**
BioGPS	biogps.org	Gene portal containing tissue distribution and eQTL data for 8 species
Gene Expression Ominbus	www.ncbi.nlm.nih.gov/geo/	NCBI repository for microarray and RNAseq data
Gene Paint	www.genepaint.org/	Tissue distribution of gene expression in the mouse embryo as determined by in situ hybridization. This includes data from the Eurexpress project.
Gene Expression Database (GXD)	www.informatics.jax.org/expression.shtml	Repository of gene expression in the mouse collected via a variety of methods
EMBL-EBI Expression atlas	https://www.ebi.ac.uk/gxa/home	Gene expression abundance and localization in multiple species including human and mouse
GeneNetwork	www.genenetwork.org/	A web service for systems genetics that includes mouse bone eQTL data and phenotype data from a large number of inbred mouse strains.

### Tissue Expression Panels

Expression data can provide information about when and where a gene is expressed. Fortunately for bone, there are excellent resources from mouse that can be used to assess tissue distribution and cell type expression. BioGPS is a gene-annotation portal that houses such data for 8 different species (84). Like many web portals, BioGPS contains a plethora of tools for easy access of the data featured. Included therein is a tissue expression panel collected by the Novartis Research Foundation. In this panel, expression of protein coding genes was assessed in a large number of primary mouse tissues from male and female C57BL/6 mice, and also from selected mouse cell lines (85), GEO Series: GSE10246). All samples were run on the Affymetrix mouse MOE430 microarray chip (GEO platform accession: GPL1261), and this data is freely available for download. Relevant to bone biology, this data set includes expression in the following cultured mouse cells from three time points post differentiation (days 5, 14, and 21), primary calvarial osteoblasts, primary cultured osteoclasts, the MC 3T3 pre-osteoblast cell line (86), the C3H10T1/2 pluripotent mouse embryonic fibroblast line, and the RAW264.7 macrophage cell line. The C3H10T1/2 cell line is considered to have mesenchymal stem cell characteristics and, if appropriately treated, these cells can be induced to become osteoblast-like, chondrocyte-like or adipocyte-like cells (87). The RAW264.7 cell line can be induced to form multinucleated, TRAP positive osteoclast-like cells (88). Thus, these three cell lines may model some features of bone stem cells. The caveat with this data is that it is microarray data, and differentiating between a lack of expression and a lack of sensitivity by the probe on the array is difficult (89). In addition to establishing that a gene is putatively expressed in bone, these data can be used to differentiate between systemic expression that would be expected for a housekeeping gene, and tissue enriched expression. Housekeeping genes are defined as genes that control basal cellular functions in most tissues and are less likely to be disease-causing genes (90). Conversely, tissue enriched genes may be informative for disease and the patterns of tissue-enriched expression may be helpful in establishing the biological role of a poorly characterized gene (91).

Newer resources for bone include data collected via next generation RNA sequencing (RNAseq). Both whole-tissue and cell type-specific expression data sets have been deposited in the public domain. Two of these data sets have been used for functional validation of GWAS loci. In the first, gene expression across osteoblastogenesis was profiled by RNAseq. In this study, primary calvarial cells were isolated from neonatal C57BL/6J mice carrying an allele whereby cyan florescent protein (CFP) expression was driven by the Col3.6 promoter (92). These cells were then sorted by FACS to remove the cells not expressing CFP and were therefore considered non-osteoblast-like. The remaining cells were placed into culture and differentiated into osteoblasts using standard protocols (93). Gene expression was measured in this osteoblast-enriched population in a dense time-course series from the pre-osteoblast to mature osteoblast stages of maturation. This is a valuable dataset for determining if a candidate gene plays a role in osteoblast maturation. Indeed this data was used to show that Engrailed 1 (*EN1*), a candidate gene for a bone mass GWAS locus, is expressed in a relevant cell type and at an appropriate time point to impact the phenotype of interest [Zheng et al. (93), GEO Series: GSE54461]. This data has been subsequently used in a number of GWAS to screen putative candidate genes (28, 33, 94, 95). The second data set was not originally created for the purpose of functionally validating GWAS loci. RNAseq data for cultured bone marrow derived mouse osteoclasts has been deposited in the Gene Expression Onmibus (GEO Accession Number: GSM1873361), and this data has been used in concert with the above osteoblast data to determine if GWAS candidate genes are expressed in relevant bone cells [28. 33]. The most abundant cell in bone tissue is the osteocyte (96) and a variety of gene expression data sets profiling expression in the osteocyte have been collected. Much of these data have not been used extensively as of yet for functional validation of human GWAS loci. Some of these data sets are so called “enrichment signature” meaning that expression is not necessarily unique to the osteocyte, but rather is higher in cells sorted based on a known osteocyte marker (97), or in a tissue type known to contain largely osteocytes (9). Using one of these data sets, Morris *et al* showed that eBMD GWAS candidate genes were highly enriched among genes showing a 4-fold higher expression in tissues high in osteocyte number as compared to bone marrow, suggesting that genes expressed in the osteocyte play a significant role in the genetic regulation of bone mass (9).

### Expression QTLs

QWAS loci overwhelmingly are thought to be caused by variants in non-coding regions (16) and may be involved in the regulation of gene expression. These variants may affect the level of transcription of the gene(s) leading to the phenotype of interest (98), or impact the post-transcriptional processing of one or more genes (99). The expression level of a gene can be used as a phenotypic trait for genetic mapping to determine if there are local alleles controlling expression. Such a locus is referred to as a *cis* expression Quantitative Trait Locus [eQTL, (100)]. Limited eQTL data exists for isolated human osteoblasts (101) and for iliac crest biopsy samples (102), but both of these data sets have low power for mapping. Use of data from other tissue types as a surrogate for expression in bone for eQTLs has yielded mixed results. This is not a unique problem for bone and, indeed, analysis of the 44 tissues collected as part of the GTEX project suggested that the distribution of the number of tissues in which a *cis*-eQTL is found is bimodal. Namely, there are a large number of eQTL found in nearly all tissues and there is an equally large number showing a high degree of selectivity in that they are found in one to three tissues only (103).

There is accumulating evidence suggesting a high degree of evolutionary conservation of patterns of gene co-expression between mice and humans in many tissues. In particular, the degree of conservation in bone is among the highest (104). Further, co-expression of pathways associated with metabolic disease, cell adhesion, and the cell cycle are also highly conserved between the species (104), suggesting conserved mechanisms of regulation. In other diseases and tissues, strong concordance for eQTL identified in mice and humans has been observed (105, 106). Collectively, this suggests that, in bone, the examination of eQTL and gene co-expression in mice would be highly informative for human bone disease and provide valuable information toward functional validation of GWAS loci. One set of data exists in the public domain that can be used for eQTL mapping in mice (GEO series number: GSE27483). This data set is comprised of long bone (sans marrow) gene expression as obtained by microarray from male mice from the Hybrid Mouse Diversity Panel (HMDP) (107). This panel of mice, which has been described in detail elsewhere (108), is comprised of 29 inbred strains, as well as 71 recombinant inbred strains of mice wherein each strain is genetically distinct. Whole body, femoral, and spinal BMD data are also available for these same strains of mice. By leveraging the genetic diversity present in this panel, loci can be mapped for both traditional and expression phenotypes (107). These phenotypic and expression data for the HMDP have been deposited in the GeneNetwork repository (http://www.genenetwork.org/). GeneNetwork is a toolbox for facilitating systems genetics (109). Deposited in the GeneNetwork repository are collections of phenotype, expression and genotype data for a number of species including mouse, rat, non-human primates, and humans. This repository is coupled to tools that facilitate analyses within a single data set or across multiple datasets. Built into GeneNetworks is the ability to conduct correlation analyses on the HMDP phenotype and genotype data, map eQTLs, and to conduct pair-scans to look for gene-gene interactions (109).

### Co-expression Networks

Network-assisted analysis of GWAS data has proven to be a powerful way to select candidate genes and provide possible mechanisms of biological action (110). The principal behind this approach is the understanding that genes function as part of larger pathways and that the allelic differences leading to complex genetic disease act on members of these pathways to mediate biological function (111). In other words, genes important for a complex disease are functionally related at some level (112). In practice, an unbiased biological network is constructed, such as a gene co-expression network (113), and the genes found in GWAS loci are mapped onto this network to identify pathways of interest and causal genes (107, 114–116). For example, bone resorption by the osteoclast is a biological function that may be perturbed in osteoporosis. There are multiple signaling pathways that control the formation and function of the osteoclast. By creating a co-expression network from bone, gene expression modules associated with this biological function of bone resorption can be identified. All genes in GWAS loci can then be overlaid to find the subset of genes that are members of these biologically relevant modules. In this manner, causal genes can be pinpointed, and biological mechanism of action is putatively determined. The important part of this method is that the networks are created in an unbiased manner as opposed to a curated or directed manner, and therefore novel discoveries can be made. Because of the conservation of co-expression between mouse and human for bone (104), network-assisted analysis of GWAS is an powerful way to augment and direct functional validation efforts for bone disease. This was elegantly demonstrated by Calbrese et al. (111). In this paper, the authors examined the 64 loci identified in the GEFOSII meta-analysis GWAS published in 2012 (117). By integrating all genes located in these 64 human loci with a gene co-expression network constructed using femoral expression data from the mouse (118), these authors were able to predict the causal gene and infer their function in bone biology for 30 of these loci. They then went on to use traditional experimental approaches to validate that two of these genes were involved in the predicted biological process and were indeed bone genes. In total, network-assisted analysis of GWAS loci is a powerful and efficient method to prioritize genes for functional validation and direct functional validation experimental design.

## Conclusions

The power of the mouse to elucidate the cause of human disease has been recognized for over 100 years. Data on gene function is being collected using mouse models at a pace and in a scope that could only be dreamed of a decade ago. In the not so distant future, a transgenic or mutant mouse model will exist for every protein coding gene in the mouse genome and with a few key strokes, any researcher, anywhere will have access to reliably collected data regarding what loss of function of that gene does to the bone and many other physiological systems. By marrying this functional data with GWAS, an unparalleled level of understanding of human disease is not over the horizon, but rather practically on our doorsteps.

The challenge moving forward will be to make sense of the function of each gene in the context of all other genes and all of the various physiological systems. Very soon it will not be adequate to write out a cell-signaling pathway as if it acted in isolation and was the sole driver of disease. As we develop new tools and methods for network analyses we are better able to comprehend and define the complex interactions leading to skeletal development, maintenance and decline. Our ultimate goal must be to determine how to leverage this new-found knowledge in the context of each person's physiology to predict, prevent or treat skeletal disease in manner that is safe and effective for that patient.

## Author Contributions

This work was written and edited by CA-B with significant writing contributions and editorial assistance from RM.

### Conflict of Interest Statement

The authors declare that the research was conducted in the absence of any commercial or financial relationships that could be construed as a potential conflict of interest.
